# Retinal Structure and Function in a Knock-in Mouse Model for the *FAM161A-*p.Arg523∗ Human Nonsense Pathogenic Variant

**DOI:** 10.1016/j.xops.2022.100229

**Published:** 2022-10-03

**Authors:** Chen Matsevich, Prakadeeswari Gopalakrishnan, Alexey Obolensky, Eyal Banin, Dror Sharon, Avigail Beryozkin

**Affiliations:** Department of Ophthalmology, Hadassah Medical Center, Faculty of Medicine, Hebrew University of Jerusalem, Jerusalem, Israel

**Keywords:** Knock-in mouse model, *Fam161a* gene, Retinal degeneration, Nonsense pathogenic variant, Disease course, ELM, external limiting membrane, ERG, electroretinography, gRNA, guide RNA, IHC, immunohistochemistry, IRD, inherited retinal degeneration, ISs, inner segments, KI, knock-in, KO, knock-out, mRNA, messenger RNA, ON, optic nerve, ONL, outer nuclear layer, OPL, outer plexiform layer, OSs, outer segments, PCR, polymerase chain reaction, PNA, peanut agglutinin, PTC, premature termination codon, RD, retinal degeneration, RP, retinitis pigmentosa, RT-PCR, reverse transcriptase-polymerase chain reaction, TRIDs, translational read-through inducing drugs, VA, visual acuity, WT, wild type

## Abstract

**Purpose:**

Pathogenic variants in *FAM161A* are the most common cause of retinitis pigmentosa in Israel. Two founder pathogenic variants explain the vast majority of cases of Jewish origin, 1 being a nonsense variant (p.Arg523∗). The aim of this study was to generate a knock-in (KI) mouse model harboring the corresponding p.Arg512∗ pathogenic variant and characterize the course of retinal disease.

**Design:**

Experimental study of a mouse animal model.

**Subjects/Participants/Controls:**

A total of 106 *Fam161a* knock-in mice and 29 wild-type mice with C57BL/6J background particiapted in this study.

**Methods:**

Homozygous *Fam161a* p.Arg512∗ KI mice were generated by Cyagen Biosciences. Visual acuity (VA) was evaluated using optomotor tracking response and retinal function was assessed by electroretinography (ERG). Retinal structure was examined in vivo using OCT and fundus autofluorescence imaging. Retinal morphometry was evaluated by histologic and immunohistochemical (IHC) analyses.

**Main Outcome Measures:**

Visual and retinal function assessments, clinical imaging examinations, quantitative histology, and IHC studies of KI as compared with wild-type (WT) mice retinas.

**Results:**

The KI model was generated by replacing 3 bp, resulting in p.Arg512∗. Homozygous KI mice that had progressive loss of VA and ERG responses until the age of 18 months, with no detectable response at 21 months. OCT showed complete loss of the outer nuclear layer at 21 months. Fundus autofluorescence imaging revealed progressive narrowing of blood vessels and formation of patchy hyper-autofluorescent and hypo-autofluorescent spots. Histologic analysis showed progressive loss of photoreceptor nuclei. Immunohistochemistry staining showed Fam161a expression mainly in photoreceptors cilia and the outer plexiform layer (OPL) in WT mice retinas, whereas faint expression was evident mainly in the cilia and OPL of KI mice.

**Conclusions:**

The *Fam161a* - p.Arg512∗ KI mouse model is characterized by widespread retinal degeneration with relatively slow progression. Surprisingly, disease onset is delayed and progression is slower compared with the previously reported knock-out model. The common human null mutation in the KI mouse model is potentially amenable for correction by translational read-through-inducing drugs and by gene augmentation therapy and RNA editing, and can serve to test these treatments as a first step toward possible application in patients.

**Financial Disclosure(s):**

The author(s) have no proprietary or commercial interest in any materials discussed in this article.

Retinitis pigmentosa [RP (OMIM #268000)] is the most prevalent inherited retinal degenerative (IRD) disease in humans, with a prevalence of 1:4500 (in Europe and USA),^1^, ^2^, ^3^, ^4^ and a higher prevalence (1:2100) in the vicinity of Jerusalem.^5^ Retinitis pigmentosa is genetically and clinically heterogeneous and can be caused by pathogenic variants in > 270 genes (RetNet, https://sph.uth.edu/retnet/), 1 of them being *FAM161A*. After the identification of *FAM161A* in 2010^6^^,^^7^ as a cause of RP, it was found to encode a ciliary protein localized to the base of the connecting cilium and is also expressed in the inner segments (ISs) of photoreceptor cells, the inner and outer plexiform layers (OPLs), and the ganglion cell layer of the retina.^8^^,^^9^ In human and mouse photoreceptor cells, FAM161A is localized to the basal body region and the adjacent centriole.^8^, ^9^, ^10^ FAM161A was shown to be part of the microtubule-organizing centers, binding directly to microtubules and increasing the acetylation of α-tubulin,^8^^,^^10^ and it also plays a role in stabilization of the cytoskeleton^8^^,^^11^ and in the assembly of the primary cilium in cell cultures.^10^ To date, 2 different mouse models for *Fam161a* were reported.^12^^,^^13^ In both models, shortened cilia with disrupted structure were documented in photoreceptor cells, indicating that Fam161a plays a role in stabilization of photoreceptor cilia as well. Thus, although its exact function remains unknown, FAM161A is important not only for ciliary structure and function in the retina, but also for additional cellular pathways.

Pathogenic variants in *FAM161A* are the most common cause for nonsyndromic RP in our Israeli cohort of > 2000 families with IRDs. More than 100 patients with biallelic autosomal recessive *FAM161A* pathogenic variants have been reported so far in the Jewish Israeli population,^14^ and all cases are explained by only 2 pathogenic variants: frameshift (c.1355_6del) and nonsense (c.1567C>T). Nonsense pathogenic variants were previously reported to make up approximately 11% to 12% of all reported pathogenic variants in genes responsible for human genetic diseases,^15^^,^^16^ and 18.5% in IRD-causing genes,^17^ although the percentage may vary among diseases, genes, and populations. In nonsense pathogenic variants, a nucleotide triplet encoding an amino acid turns into a premature termination codon (PTC) that causes an early termination of the protein and results in a shorter, usually nonfunctional, peptide. In approximately 0.01% to 1% of the cases, spontaneous translational read-through can occur and insert a near cognate transfer RNA, which will result in insertion of an alternative amino acid (equivalent to missense variant) and skipping of the PTC.^18^^,^^19^

Recently, several potential strategies for enhancing PTC skipping have emerged, 1 of them being the use of translational read-through inducing drugs (TRIDs). Translational read-through inducing drugs include aminoglycoside antibiotics, their derivatives, and also small molecules that are able to overcome the PTC and induce the translational read-through process. This results in translation of a full-length protein with a single amino acid change, but probably active, protein.^20^, ^21^, ^22^, ^23^ We recently reported that treatment with different TRIDs has a positive effect on ciliogenesis, cilia length, and localization of FAM161A along microtubules of ciliated fibroblast cells that originated from *FAM161A*-RP patients harboring the common nonsense pathogenic variant in a homozygous state.^24^

In the present study, we describe the generation and course of disease in a new knock-in (KI) mouse model for *Fam161a* carrying the homozygous nonsense pathogenic variant p.Arg512∗ (NM_001363282), which is equivalent to the human pathogenic variant p.Arg523∗. Our goal in future studies is to use this model for testing different therapeutic approaches in vivo, including TRIDs.

## Methods

Experiments were conducted in compliance with the Association for Research in Vision and Ophthalmology Statement for the Use of Animals in Ophthalmic and Vision Research and were approved by the Hebrew University animal ethics committee.

### *Fam161a* KI Mouse Model Generation

Mice were generated by Cyagen Biosciences (www.cyagen.com) using CRISPR/Cas9. The KI pathogenic variant was created in exon 3 of *Fam161a* using the following guide RNAs (gRNA): gRNA1 (matches the forward strand): CCACCGCGTCTTCCCGAGGGCGG, and gRNA2 (matches the reverse strand): GATGGCTTGCTCCCGCCCTCGGG (Fig S1A, available at https://www.ophthalmologyscience.org). Analyses of potential off-target sites were performed. Off-target analysis for gRNA1 revealed 63 sites, 21 of them in genes. The genes with the highest probability to be affected by CRISPR/Cas9 manipulation are *Xrn2*, *Crlf1*, *Sptbn4*, *Lbx2*, *Sec31b*, *Ogfod3*, *Gimap8*, *Scpep1os*, and *Nle1*. Off-target analysis for gRNA2 revealed 116 sites, 21 of them in genes. The genes with the highest probability to be affected by CRISPR/Cas9 manipulation are *Thbd*, *Lrrc52*, *Ptprs*, *Syt16*, *Mroh8*, *Dot1l*, *Ncoa6*, and *Ptk2*. None of those genes are expected to cause IRD. Guide RNA targeting vector and donor oligo (with targeting sequence, flanked by 130 bp homologous sequences combined on both sides) were designed. The p.Arg512∗ (CGG to TGA) mutation sites in donor oligo were introduced into exon 3 by homology-directed repair. Cas9 messenger RNA (mRNA), gRNA generated by in vitro transcription, and donor oligo were coinjected into fertilized eggs of C57BL/6J mouse for KI mouse production.

Mice received from Cyagen were heterozygous for the KI mutation. Those mice were genotyped by polymerase chain reaction (PCR) followed by sequence analysis and screened for known IRD pathogenic variants in *Crb1* (rd8),^25^ *Rpe65* (rd12),^26^ *Gnat2*,^27^ *Pde6b* (rd10),^27^ and (rd1)^28^ using Sanger sequencing (Table S1, available at https://www.ophthalmologyscience.org). Ten generations of heterozygous *Fam161a* mice were crossed with C57BL/6J mice to establish a strain that has a clear and identical background. The last generation was intercrossed to establish homozygous KI and wild-type (WT) (without a mutation) mice with identical background. Wild-type mice were used as controls, whereas heterozygous mice were excluded.

### Mice Maintenance and Treatment

Animals were maintained at the animal facility of the Hebrew University-Hadassah Medical Center, Ein-Kerem, Jerusalem, Israel, and kept under specific pathogen-free and 12-hour light/dark conditions. General microbial examinations were routinely performed by the animal research facility staff. The weight, development, growth, and behavior of the KI animals were also routinely checked and seemed to be normal.

Retinal structure and function were studied at the ages of 1, 3, 6, 9, 12, 18, and 21 months. Mice were anesthetized according to standard protocol as previously described.^13^ Pupils were dilated with 1% tropicamide and 2.5% phenylephrine, local anesthetic drops were administered (benoxinate hydrochloride, 0.4%; all ocular drops from Fisher Pharmaceuticals), and eyes were lubricated with methylcellulose before electroretinography (ERG), fundus photography, and OCT imaging as detailed later.

### Genotyping

DNA was extracted from mice ear punches using 180 μl of 50 mM sodium hydroxide per 5 to 10 mg of mouse ear sample and incubated for 10 minutes at 95° C. Extract was neutralized by adding 20 μl of 1M Tris- hydrochloride (pH 8.0). Genotyping was performed using KAPA Mouse Genotyping Kit (KAPA Biosystems). Genotyping primers targeting the WT or KI alleles are reported in Table S1 (available at https://www.ophthalmologyscience.org).

### RNA Isolation and RT-PCR Analysis

The retina was dissected out of the eye and immediately placed in TRI reagent on ice (T9424, Sigma-Aldrich). RNA was extracted from mouse retinas using TRI Reagent following the manufacturer’s protocol (https://www.sigmaaldrich.com/US/en/technical-documents/protocol/protein-biology/protein-lysis-and-extraction/tri-reagent). RNA was converted to complementary DNA using the qScript complementary DNA synthesis kit (PSF-95047-100, Quanta bio) following the protocol provided by the company (https://www.quantabio.com/product/qscript-cdna-synthesis-kit/). Reverse Transcriptase-Polymerase chain reaction (RT-PCR) was performed with 50 ng of complementary DNA to amplify exons 3 to 5 (Table S1, available at https://www.ophthalmologyscience.org).

### Optomotor Tracking Response

Visual acuity (VA) was evaluated using an optomotor testing apparatus (OptoMotry; Cerebral Mechanics, Inc) by recording the tracking response (optomotor reflex) of the head to a rotating visual stimulus displayed on 4 LCD panels surrounding the awake mouse. This is a common method for estimating VA in mice and has previously been described and evaluated in detail.^29^ The results are presented in cycles/degree that is equal to the number of cycles of a grating (1 dark and 1 light vertical line) that subtends an angle of 1° at the mouse eye. This test is demonstrated in a short video (Supplementary Video S1, available at https://www.ophthalmologyscience.org). Visual acuity was measured at 100% contrast in 8 to 14 mice per age group. Wild-type mice were tested at the ages of 1 month (n = 8) and 20 months (n = 7). Knock-in mice were tested at the ages of 1 month (n = 14), 3 months (n = 14), 6 months (n = 12), 9 months (n = 12), 12 months (n = 13), 15 months (n = 10), 18 months (n = 8), and 21 months (n = 8).

### ERG

Full field ERG was performed on anesthetized animals (n = 9–17 per age group) after overnight dark adaptation. Wild-type mice were tested at the ages of 1 month (n = 10) and 20 months (n = 7). Knock-in mice were tested at the ages of 1 month (n = 10), 3 months (n = 17), 6 months (n = 16), 9 months (n = 17), 12 months (n = 13), 15 months (n = 9), 18 months (n = 11), and 21 months (n = 10). Full field ERG was performed using a Ganzfeld dome and a computerized system (Espion E2, Diagnosys LLC), as previously described.^30^ Briefly, pupils were dilated, and gold-wire active electrodes were placed on the central cornea. A reference electrode was placed on the tongue and a needle ground electrode was placed intra-muscular (IM) in the hip area. Dark-adapted rod and mixed cone-rod as well as light-adapted 1 Hz and 16 Hz cone flicker responses to a series of white flashes of increasing intensities (0.00008–9.6 cd·s/m^2^) were recorded. All ERG responses were filtered at 0.3 to 500 Hz, and signal averaging was applied. All mice that underwent ERG examination (31 KI and 12 control WT mice) were also serially evaluated by OCT imaging.

### OCT, Infrared, and Fundus Autofluorescence Imaging

Retinal structure was studied in vivo using single horizontal 30° OCT b-scans passing through the optic nerve (ON), and by infrared and fundus autofluorescence imaging (SPECTRALIS, Heidelberg). OCT is a noninvasive technology used to obtain cross-sectional images of the retina with very high resolution, including assessment of the presence and thickness of the outer nuclear layer (ONL) (photoreceptor nuclei layer), which is progressively lost during the course of retinal degeneration (RD). Two additional methods, infrared and fundus auto-fluorescence imaging, also enable the evaluation of retinal changes that develop and progress during degeneration, especially the development of abnormalities in the retinal pigment epithelium, often followed by development of chorioretinal atrophy.

The procedures were performed in anesthetized animals with dilated pupils and lubricated eyes as mentioned earlier. At least 10 animals were examined in each age group. The retina of each mouse that participated in the study was imaged by a single 30° OCT b-scan through the ON, which allowed the uniformity of the area studied, the ability to perform serial imaging at the same location, and enabled the evaluation of whether the degeneration is symmetric. Images are presented sequentially to compare and show follow-up of the RD phenotype. Serial images of 1 WT mouse at 2 timepoints (1 and 20 month/s) and 1 KI mouse at the ages of 1, 3, 6, 9, 12, 15, 18, and 21 month/s are presented. Serial images of a control WT mouse at the same full range of ages are presented in Figure S2 (available at https://www.ophthalmologyscience.org).

### In Vitro Evaluation of Retinal Structure

Histology and Retinal morphometry were performed on another group of mice. Because this is a terminal procedure, a total of 75 KI mice and 17 WT mice were raised for this purpose, and at each timepoint ≥ 8 mice were euthanized and their eyes analyzed. The KI group included mice at the age of 1 month (n = 10), 3 months (n = 11), 6 months (n = 9), 9 months (n = 10), 12 months (n = 9), 15 months (n = 10), 18 months (n = 8), 21 months (n = 8), and the WT group included mice at the ages of 1 month (n = 10) and 20 months (n = 7).

### Histology and Immunohistochemistry

Eyes were enucleated, fixed in Davidson solution for 8 hours at 4° C, and moved to 70% ethanol over-night at 4° C. Eyes were then incubated in a 80% -> 90% -> 100% -> 100% ethanol gradient at room temperature for 30 minutes per each concentration, placed for 15 minutes in ethanol 100%:xylene (1:1) solution at room temperature, and washed twice with xylene 100% for 20 minutes per wash. Samples were incubated in paraffin (Paraplast Plus, Leica Biosystems) in 58 °C for 3 incubations, each lasting 40 minutes. Later, they were embedded in paraplast and serially cut into 5-μm thickness sections through the center of the ON. For immunohistochemistry (IHC), a standard immunohistologic protocol for paraffin sections was carried out, as previously described.^31^ Double labeling and double staining were carried out according to a protocol previously described.^32^ Commercial buffers (ImmunoRetriever 20× with citrate pH 6.62, Bio Sb or Antigen Retrieval Buffer -100× Citrate Buffer pH 6.0) were used. The following commercial antibodies were used: anti-Fam161a (HPA032119, Sigma-Aldrich) to identify the Fam161a protein, Anti-Peanut Agglutinin (PNA) (FL-1071-5, Vector laboratories) to recognize both types of mouse cone photoreceptors, and Antiblue Opsin (SC-16363) and antired/green Opsin (AB5405, Millipore Sigma) antibodies were used to recognize the subpopulations of cone cells in the retina. Anti-Rhodopsin (MS-1233P, Thermo Fisher) allowed for the identification of rod photoreceptor cells. Negative control was exposed to secondary antibody only (without previous exposure to primary antibody) to demonstrate the specificity of the secondary antibody.

Retinal morphometry was assessed in histologic sections passing through the ON following staining with hematoxylin–eosin according to standard protocol.^30^ To quantify thickness at different eccentricities, 150 μm steps were measured from the ON toward the nasal and temporal periphery, and total retinal thickness as well as thickness of the ONL and number of rows of nuclei in the ONL were measured separately in each 150-μm section by ImageJ software. Comparison between ONL thickness in different *FAM161a* models (*Fam161a*^GT/GT^,^12^ *Fam161a*^tm1b/tm1b^,^13^ and the current study model *Fam161a* KI) was conducted using the average number of nuclei rows in each timepoint per each model and based on the data that were previously published in the aforementioned articles.

Student *t* test was performed to determine the significance of the observed differences in total retinal thickness, ONL thickness, and number of rows of nuclei in the ONL (unpaired, 2-tailed, assuming equal variance). The total retinal thickness and ONL thickness of 20-month WT mice was compared with the thickness of 1-month WT mice. The same measurements were compared between 1-month-old WT and 1-month-old KI mice. Older KI mice were compared with the results obtained from the same group in the previous timepoint (e.g., 6-month-old KI compared with 3-month-old KI). In addition, we compared older KI mice with the results obtained from a group of 1-month KI animals, until the first timepoint in which statistically significant difference was found. Significance levels were set at ∗*P* value of < 0.05, ∗∗*P* value of < 0.01, and ∗∗∗*P* value of < 0.001.

### Evaluation of Cone Photoreceptors Survival

A single image of × 40 magnification of the nasal part of the mid periphery of the retina was taken from WT mice at the age of 1 month (n = 8) and 6 months (n = 6) and from KI mice at the ages of 1 month (n = 7) and 6 months (n = 6). Cells positive for PNA, blue opsin, or red/green opsin were counted in every image. Student *t* test was performed to test the significance of the observed differences in the number of the positive cells among the different groups (unpaired, 2-tailed, assuming equal variance). The total number of positive cells for a certain staining was compared between WT and KI mice at every timepoint. In addition, the total number of positive cells for a certain staining was compared between 1 month and 6 months for each mouse strain. Statistically significant levels were set at ∗ *P* value of < 0.05, ∗∗ *P* value of < 0.01, and ∗∗∗*P* value of < 0.001.

### Histologic and IHC Imaging

All observations and photography of retinal sections were performed using an Olympus BX41 microscope equipped with a DP70 digital camera. Confocal images were captured using a ZEISS LSM980 with Airscan microscope. Image processing and quantification were performed using Adobe Photoshop CS2, Canvas X, and ImageJ software.

### Data Availability

The datasets generated and analyzed in the current study are available from the corresponding authors on reasonable request.

## Results

### Generation of *Fam161a* KI mice

We generated a homozygous mouse line with targeted insertion-deletion, in which the triplet TGA was inserted instead of CGG in *Fam161a*- exon #3 using CRISPR/Cas9 (Fig S1A, available at https://www.ophthalmologyscience.org), resulting in p.Arg512∗ (NM_001363282). This nonsense pathogenic variant was verified using Sanger sequencing of PCR products (Fig S1B, available at https://www.ophthalmologyscience.org). *Fam161a* KI founders were screened and found negative for other IRD pathogenic variants common in mouse colonies.

Aiming to study the effect of the pathogenic variant at the RNA level, we performed RT-PCR analysis on *Fam161a* KI and WT retina at the age of 1 month using primers located in exons 3 and 5 (Table S1, available at https://www.ophthalmologyscience.org). Agarose gel analysis revealed the expression of both transcripts (including the skipping of exon #4) in KI and WT mice samples, indicating that the mutant transcript is not degraded (Fig S1C, available at https://www.ophthalmologyscience.org). The sequence of all bands was verified by Sanger sequencing.

### The Effect of p.Arg512∗ on Fam161a Expression and Localization

**Figure 1:**
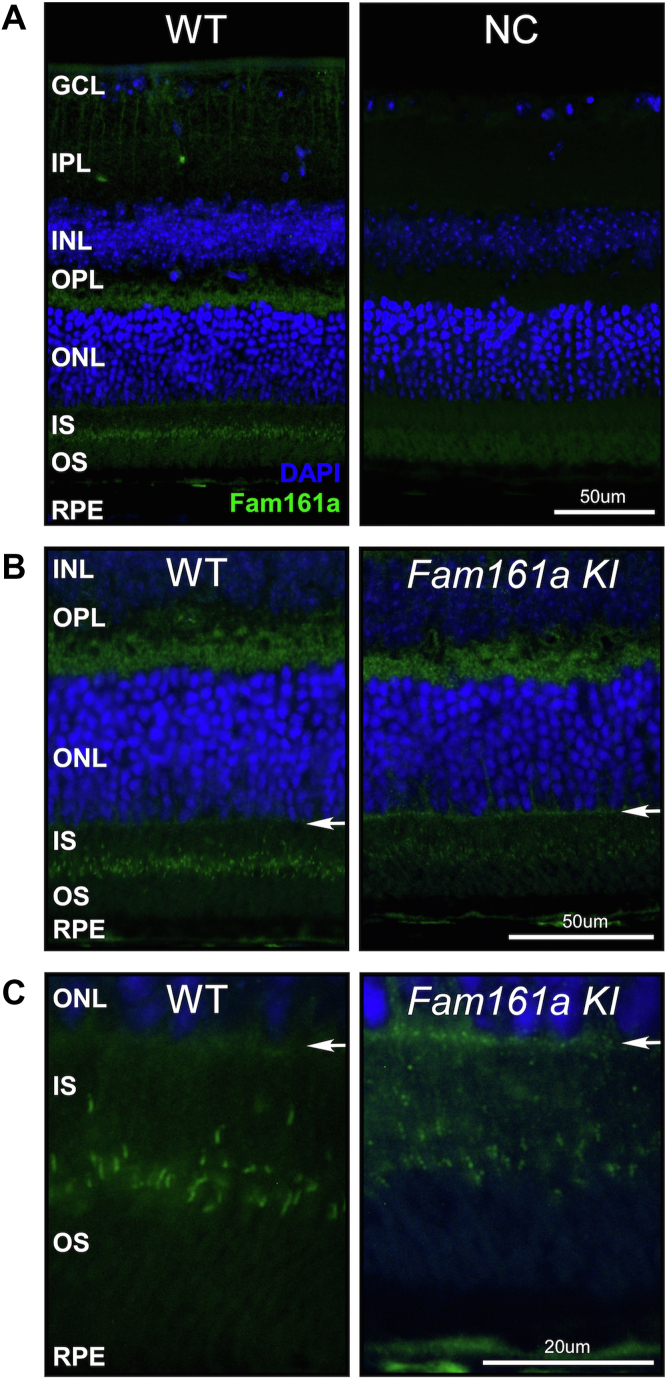
Fam161a expression in the retina. **A,** immunohistochemical (IHC) images of 1-month old wild-type (WT) mouse retina, with (left panel) and without (right panel) primary rabbit anti-Fam161a antibody (HPA032119, Sigma) against Fam161a (in green). The retina was counterstained with 4′,6-diamidino-2-phenylindole (DAPI) to define nuclear layers. (IHC stain, Fam161a: green, DAPI blue; original magnification, × 20). **B,** IHC images from 1 month old WT (left panel) and knock-in (KI) (right panel) retinas stained with anti-Fam161a antibody (green) and DAPI (blue). Scale bar = 50 μm. (stain; original magnification, × 40). **C,** Stain; higher magnification, × 100 of the same sections. Scale bar = 20 μm. GCL = ganglion cell layer; IPL = inner plexiform layer; INL = inner nuclear layer; NC = negative control; OPL = outer plexiform layer; ONL = outer nuclear layer; IS = inner segments; OS = outer segments; RPE = retinal pigment epithelium. White arrows point to the external limiting membrane.

To determine the expression pattern of Fam161a in the mouse retina and how it is affected by the p.Arg512∗ pathogenic variant, we used IHC staining. Staining of a WT mouse retina with a rabbit anti-Fam161a antibody revealed expression of Fam161a mainly between the inner and outer segments (ISs∖OSs) of photoreceptors and in the OPL (Fig 1A–C, left panels), whereas no staining was evident in the negative control, which was exposed to the secondary antibody only (Fig 1A, right panel). In addition, relatively low expression levels can be seen along the external limiting membrane (ELM) and between the nuclei of the ONL toward the ELM in the WT retina (Fig 1, left panels). Performing the same staining in a KI retina at the same age (1-month old) revealed lower expression with an abnormal pattern (Fig 1B, C, right panels): very low and diffuse expression in the ISs∖OSs region, relatively intense expression in the ELM, and an abnormal expression pattern in the OPL. These results indicate that in the KI retina, the mutant Fam161a protein is mislocalized and is unable to reach its target.

### Visual Function of *Fam161a* KI Mice

**Figure 2:**
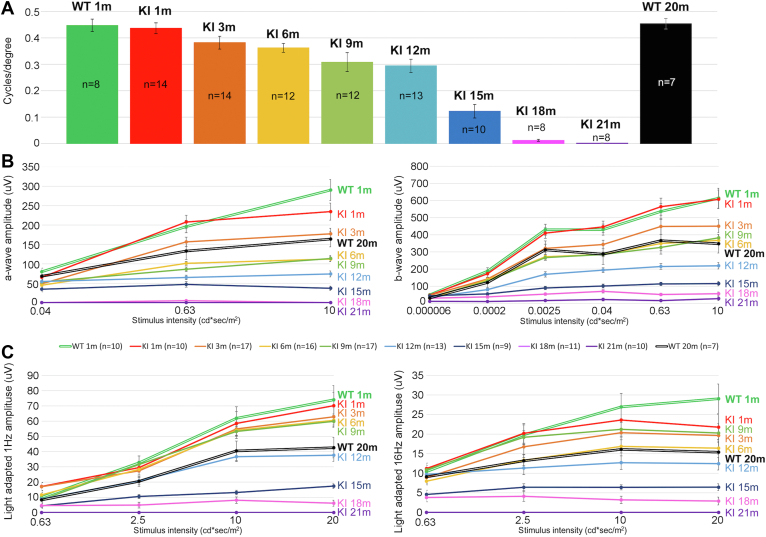
Retinal function in *Fam161a* KI mice compared with wild-type (WT) mice as demonstrated by assessment of optomotor tracking response and electroretinography (ERG). **A,** Average results of visual acuity in WT and knock-in (KI) mice. The number of tested mice is written inside each bar. WT group consists of 8 mice at the age of 1 month and 7 mice at the age of 20 months. Different colors represent different ages. **B,** Scotopic a-wave and b-wave ERG amplitudes of WT at the age of 1 (green, bold double line) and 20 months (black, bold double line) and *Fam161a* KI at the ages of 1, 3, 6, 9, 12, 15, 18, and 21 months (red, orange, yellow, light green, light blue, dark blue, pink and purple, respectively). **C,** ERG responses under photopic conditions (1 Hz and 16 Hz stimulus) of WT and *Fam161a* KI mice at the same ages. Error bars in **A** and **B** show standard error of the mean.

Visual acuity in *Fam161a* KI and in control WT mice was estimated by assessing optomotor responses (head movements in response to rotating visual stimulation). At the age of 1 month, VA in KI mice was slightly reduced compared with WT mice at the same age (by 3%), a difference that was not significant (*P* = 0.05) (Fig 2A). During the follow-up examinations over time, VA deteriorated in KI mice, while remaining stable in WT mice until the last timepoint at 20 months of age. Visual acuity of mice at the age of 3 months was reduced by 12.5% compared with the previous timepoint. Up to the age of 12 months, the deterioration in VA was a decrease of 5% to 15% between every 2 adjacent timepoints. The most rapid deterioration occurred between the ages of 12 and 15 months, during which KI mice lost > 58% of their previously measured VA. At 18 months of age, approximately half of the KI mice did not respond to changes between dark and light screens, and at the age of 21 months, none of them responded (Fig 2A).

Retinal function was further assessed by ERG recordings. Scotopic and photopic responses were measured every 3 months. In WT mice, natural deterioration in scotopic and photopic responses was detected over time such that at the age of 20 months (black bold double line; Fig 2B, C), amplitudes were approximately 50% of those recorded at 1 month of age (green bold double line; Fig 2B, C). In KI mice, scotopic and photopic amplitudes deteriorated more rapidly compared with WT mice, although more slowly than in other commonly used mouse models of RD, such as rd10 mice^33^ or *Fam161a* knock-out (KO) model. At the age of 1 month, no or borderline significant difference was observed between KI and WT mice. A more pronounced gradual slow deterioration with increasing differences between KI and WT animals is evident starting at 3 months of age, and by the age of 21 months ERG responses were nondetectable in KI mice, but still quite robust in WT animals (Fig 2B, C). The decrease in scotopic amplitudes was more prominent during the first months (reduction of 20%–25% in the scotopic amplitude between each timepoint until the age of 6 months, compared with 7%–14% reduction in photopic amplitudes), mimicking the rod>cone course of disease in human patients. For example, at the age of 18 months low-photopic responses are still evident, whereas scotopic a-waves are nondetectable, and scotopic b-waves are barely detectable. In addition, in correlation with the VA assessments, photopic 1 Hz responses in KI mice dropped dramatically between the age of 9 and 15 months.

### Retinal In Vivo Imaging of *Fam161a* KI Mice

**Figure 3:**
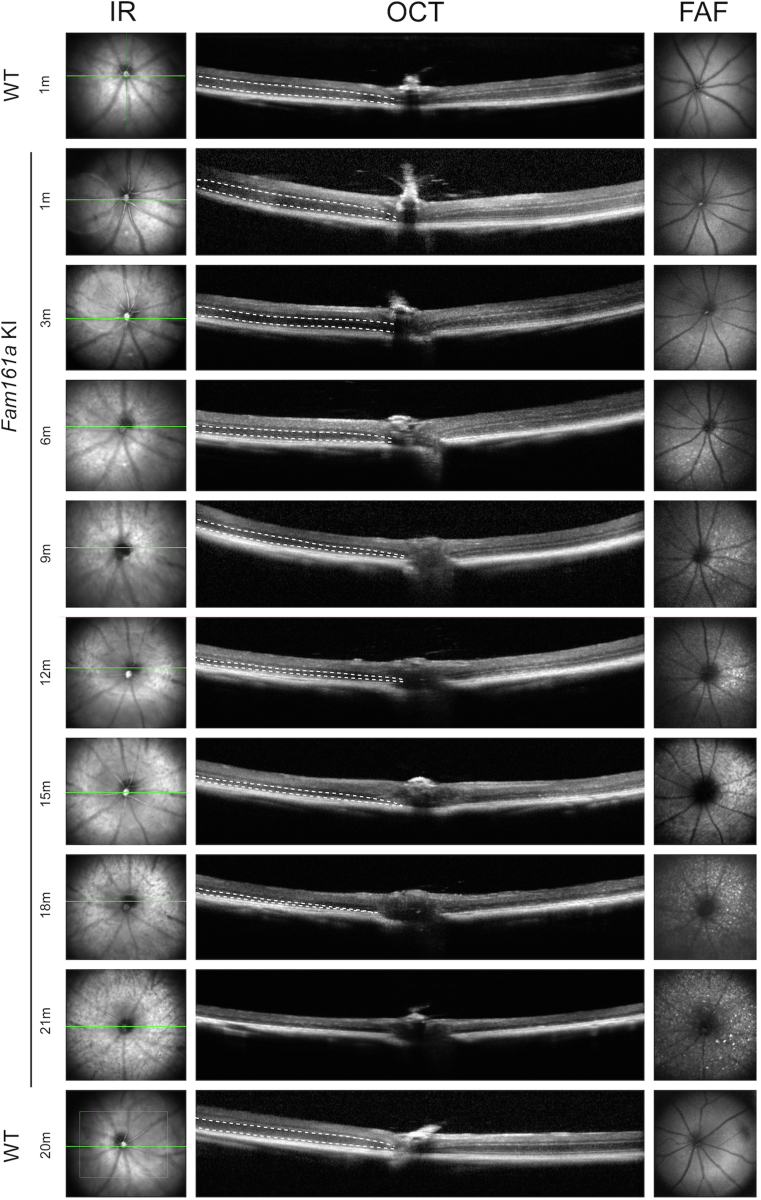
Infrared (IR), OCT and fundus auto-fluorescence (FAF) imaging of *Fam161a* knock-in (KI) mouse retinas at different ages. A comparison between *Fam161a* KI mouse retinas at 1, 3, 6, 9, 12, 18, and 21 month/s of age and wild-type (WT) mouse retina at the ages of 1 and 20 months is presented. The outer nuclear layer (ONL) that represents the photoreceptors is marked by dotted lines. Note gradual thinning of the ONL over time in KI, becoming nondetectable at 21 months of age.

**Figure 4:**
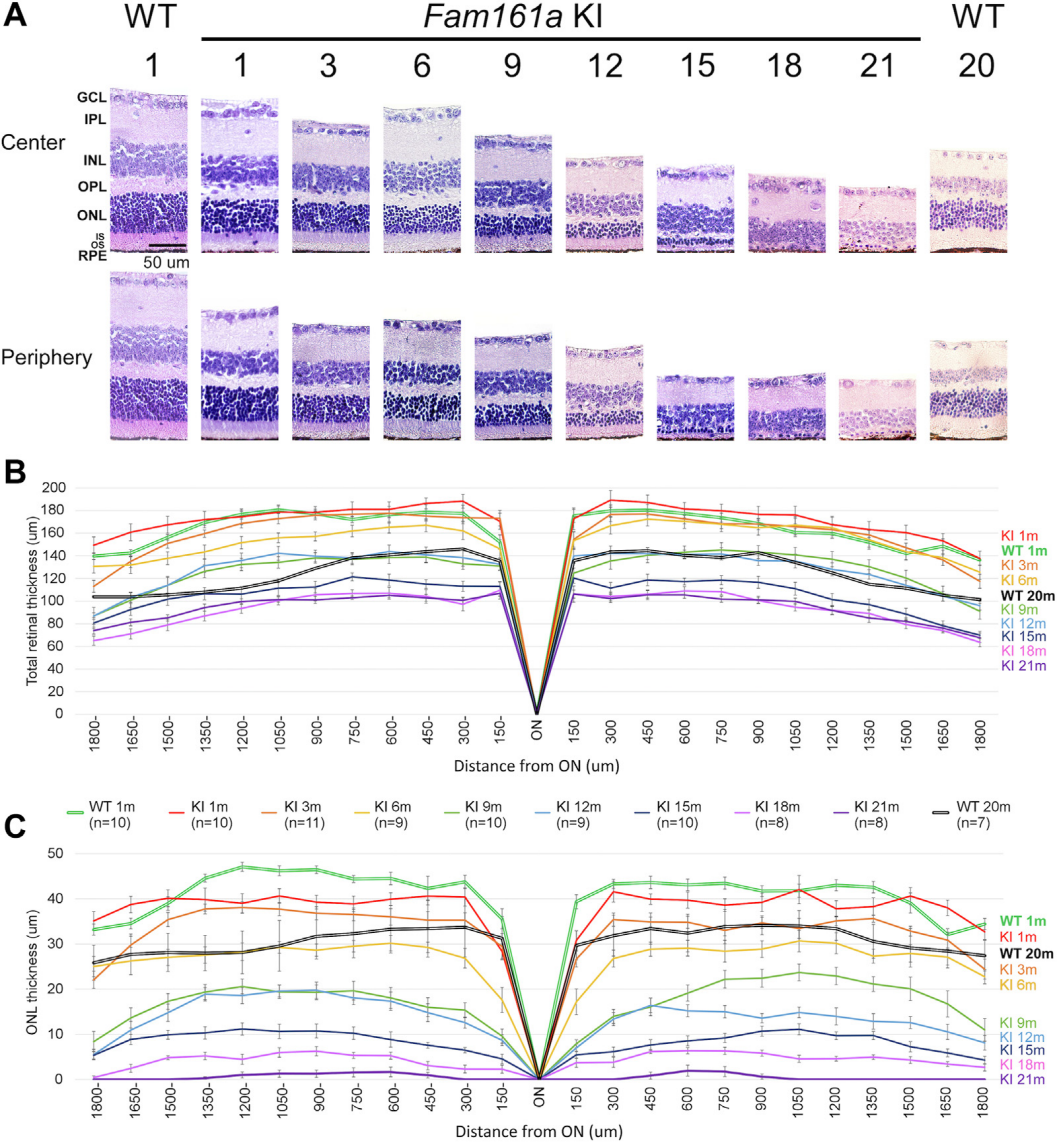
Retinal structure in *Fam161a* knock-in (KI) mice compared with wild-type (WT) mice as demonstrated by histologic analysis. **A–C**, Progressive degeneration of the retina in *Fam161a* KI mice (total number of mice at all timepoints equals 75) compared with 1- and 20-month old WT mice (n = 17) as reflected by retinal structure and thickness. **A,** Representative images at different ages from central retina and far-periphery retina stain, hematoxylin–eosin; original magnification, × 40). Scale bar = 50 μm. **B,** Total retinal thickness in *Fam161a* KI versus WT mice. **C,** Outer nuclear layer (ONL) thickness in *Fam161a* KI and WT mice. Error bars represent standard error of the mean. GCL = ganglion cell layer; INL = inner nuclear layer; IPL = inner plexiform layer; ON = optic nerve; OPL = outer plexiform layer; RPE = retinal pigment epithelium.

In vivo imaging analyses of retinal structure and fundus appearance were performed at the same timepoints in which retinal function was measured. Fundus autofluorescence imaging revealed narrowing of blood vessels and formation of patchy hypo-autofluorescent and hyper-autofluorescent spots in the retina that were first clearly observed at the age of 3 months in KI mice. These spots became more prominent over time, indicating widespread RD in KI mice (Fig 3). Such changes were not observed in WT mice even at the age of 20 months, indicating a healthy retina (Fig S2, available at https://www.ophthalmologyscience.org). OCT images that were uniformly taken through the ON showed gradual thinning of the ONL over time in KI mice. Finally, at the age of 18 months, the ONL could no longer be identified on OCT scans, and at the age of 21 months, the OPL was also practically absent. Retinal thickness in WT mice was slightly thinner at the age of 20 months than at the age of 1 month (Fig S2, available at https://www.ophthalmologyscience.org; results that are consistent with the literature^34^), but much better preserved than 21-month KI mice (Fig 3). These results correspond to the histologic retinal findings described later (Fig 4A).

### Retinal Structure of *Fam161a* KI Mice

Histologic retinal sections of 75 *Fam161a* KI and 17 WT mice (as detailed in the methods section) were stained with hematoxylin–eosin and the thickness of the retinal layers was measured using ImageJ software. The sections, all of which go through the ON for uniformity, demonstrate gradual thinning of the retina, mainly the ONL in *Fam161a* KI, mice over time (Fig 4A–C).

We measured the ONL and total retinal thickness in WT mice at the ages of 1 and 20 months and in KI mice at 1, 3, 6, 9, 12, 18, and 21 month/s of age (7–11 mice in each group). In WT mice, total retinal thickness decreased slightly between 1 and 20 months of age; however, even at 20 months, a relatively well-preserved ONL was evident, with ∼ 9 rows of photoreceptor nuclei in proximity to the ON (Fig 4A–C, Table 1, and Fig S3, available at https://www.ophthalmologyscience.org). In KI mice, mild thinning of the ONL was already evident at the age of 1 month (loss of ∼ 1 row of nuclei compared with WT), and progressive thinning of the whole retina and mainly the ONL continued until the age of 21 months, when only few photoreceptor nuclei remain. The decrease in total retinal thickness became statistically significant at the age of 9 months (Fig 4B and Table 1), while thinning of the ONL became statistically significant at the age of 6 months (Fig 4C and Table 1). At the age of 9 months, KI mice lost approximately half of their photoreceptor nuclei layer, and at the age of 21 months, only several remaining nuclei in the ONL were evident (Fig S3, available at https://www.ophthalmologyscience.org). The degeneration of the cells within the ONL explains most of the loss of total retina thickness.

**Table 1:** Total Retinal and Outer Nuclear Layer Thickness in WT and *Fam161a* KI Mice

Genotype	WT		*Fam161a* KI							
Age (mos)	1	20	1	3	6	9	12	15	18	21
Total retinal thickness (mean, μm) *P* value	164.78	125.94∗∗∗	172.26 0.48	160.75 0.29	154.18 0.53	127.93∗∗	128.8 0.92	105.94∗∗	93.93 0.1	95.05 0.86
Outer nuclear layer thickness (mean, μm) *P* value	41.21	30.89∗∗∗	38.33 0.27	33.41∗	27.1∗∗	17.24∗∗∗	13.87∗	8.34∗∗∗	4.36∗∗∗	0.54∗∗∗
Outer nuclear layer thickness (mean, # of nuclei) *P* value	10.41	8.32∗∗∗	9.77 0.34	7.99∗∗	5.56∗∗∗	3.48∗∗∗	2.76∗	1.88∗∗∗	1.22∗∗∗	0.14∗∗∗

KI = knock-in; WT = wild type.

∗Significance levels were set when *P* < 0.05.

∗∗*P* < 0.01.

∗∗∗*P* < 0.001.

Comparison of ONL thickness between 2 previously published mouse models (*Fam161a*^GT/GT^,^12^ and *Fam161a*^tm1b/tm1b^,^13^) and the current *Fam161a* KI model was performed. Reduction in ONL thickness was evident in all mouse models. The reduction is slightly faster in the *Fam161a*^GT/GT^ model than that in the *Fam161a*^tm1b/tm1b^ model, because the *Fam161a*^GT/GT^ mice lose ∼ 90% of the ONL thickness 2 months earlier than *Fam161a*^tm1b/tm1b^ mice, 6 months as compared to 8 months of age, respectively. The current *Fam161a* KI model is much slower and reaches the same degree of ONL thickness loss > 1 year after the faster of the 2 models (Fig S4, available at https://www.ophthalmologyscience.org).

**Figure 5:**
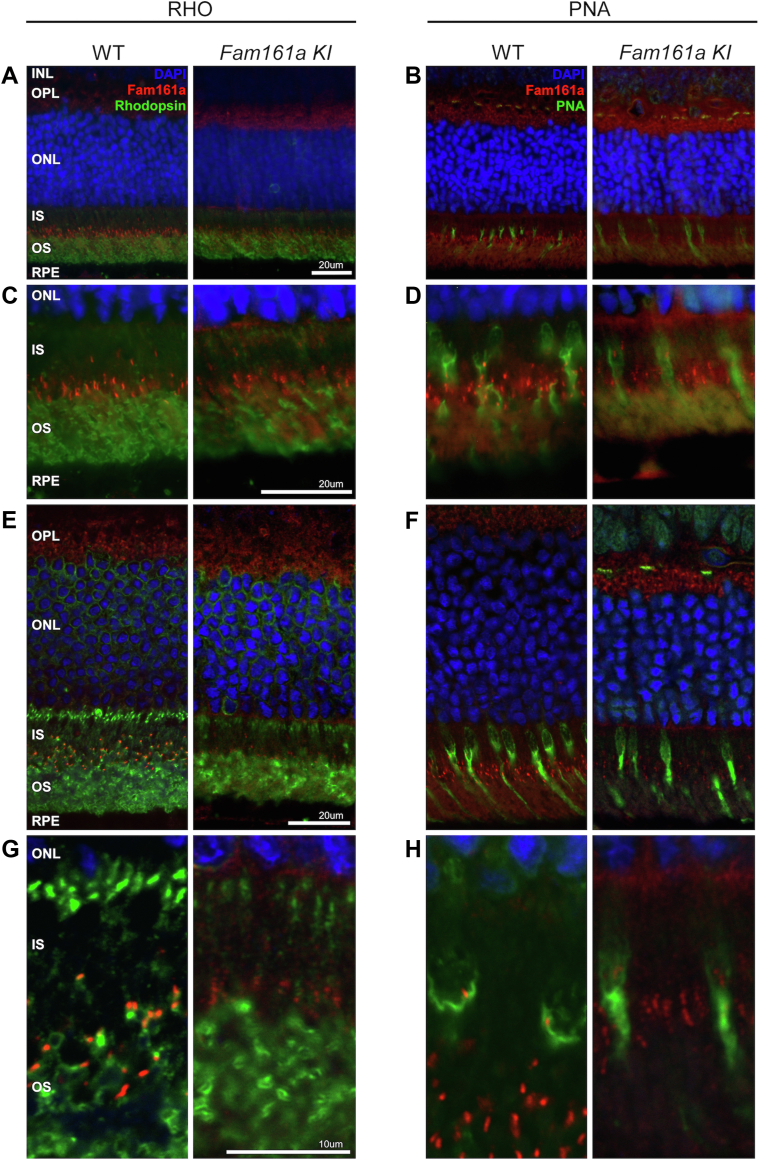
Fam161a expression in different types of photoreceptors. **A,** Representative immunohistochemistry (IHC) images from 1-month-old wild-type (WT) and 1-month-old *Fam161a* knock-in (KI) mice retina, with primary rabbit anti-Fam161a antibody against Fam161a (red) and primary mouse anti-Rhodopsin (RHO) antibody (green). Retinas were counterstained with 4′,6-diamidino-2-phenylindole (DAPI) (blue) to define nuclei. (IHC stain; original magnification, ×40. Scale bar = 20 μm. **B,** Images from 1-month-old WT and 1-month-old KI mice retina, with primary rabbit anti-Fam161a antibody against Fam161a (red), anti-Peanut Agglutinin (PNA) (green) and DAPI (blue). (IHC stain; original magnification, × 40). Scale bar = 20 μm. **C,** IHC stain; higher magnification, × 100 of the staining presented in **A**. Scale bar = 20 μm. **D,** IHC stain; higher magnification, ×100 of the staining presented in **B**. Scale bar = 20 μm. **E–H,** The same staining captured with a confocal microscope. **E, F,** IHC stain; original magnification, × 63. Scale bar = 20 μm. **G**, **H,** IHC stain; higher magnification of the previous panels, with additional × 4 magnification. Scale bar = 10 μm. IS = inner segments; INL = inner nuclear layer; ONL = outer nuclear layer; OPL = outer plexiform layer; OS = outer segments; RPE = retinal pigment epithelium.

To estimate the expression of Fam161a in rod and cone photoreceptors, we performed double IHC staining of either Rhodopsin or PNA antibodies together with Fam161a (Fig 5). The results clearly show that Fam161a is located along the cilia of both types of photoreceptors. Rhodopsin was used as a specific marker for rod OSs and Fam161a expression was evident along the cilia between the ISs and OSs of rods in WT retinas (Fig 5A, C). Interestingly, the expression of Rhodopsin in the KI retina is different from the 1 observed in the WT (Fig 5A, C, E, G), probably because of rod degeneration. It was also evident that Fam161a expression is not restricted only to rods, but is also expressed along the cilia of cones, which were immunostained with anti-PNA antibody (Fig 5B, D, F, H). Staining of the KI mouse retina showed faint and aggregated expression of Fam161a in the photoreceptors of both rods and cones and along the ELM and at the base of the ISs (Fig 5A–D). Confocal microscopy images show this pattern in higher resolution (Fig 5E–H).

**Figure 6:**
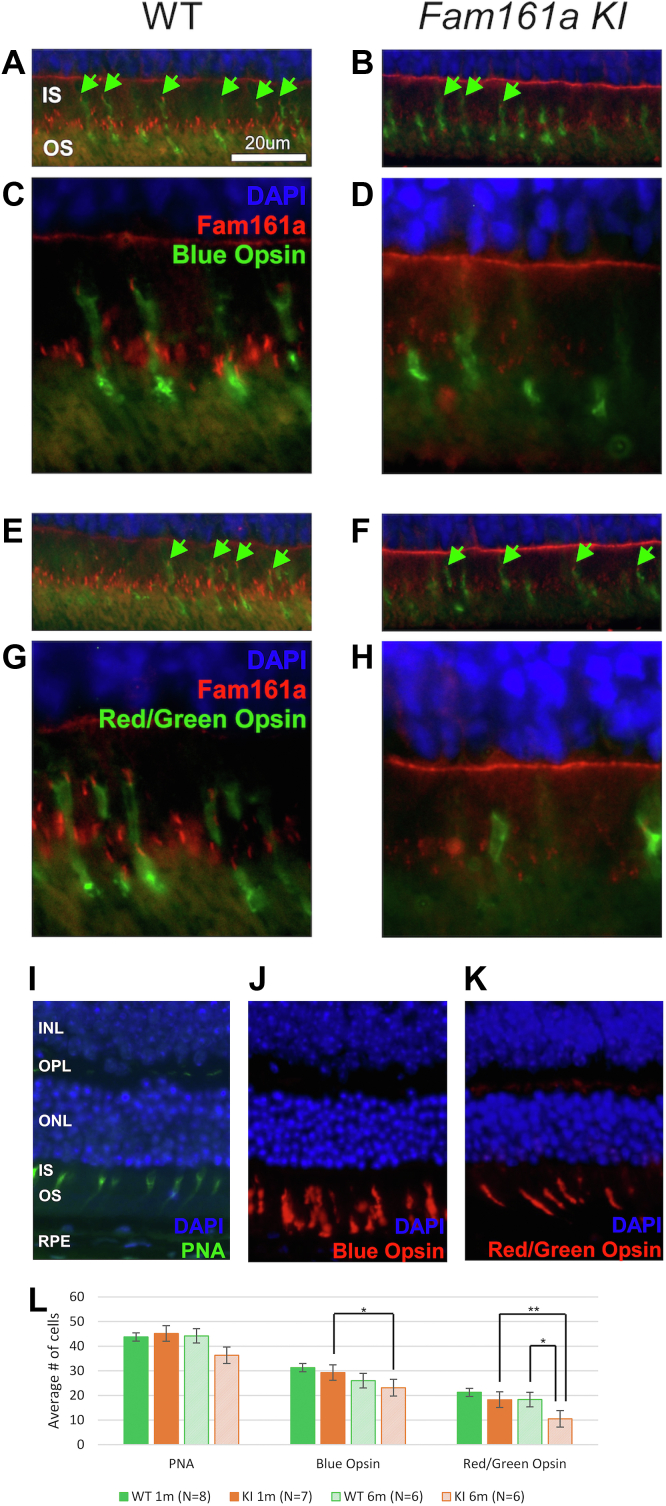
Fam161a expression in different types of cones. Double staining of 1-month-old wild-type (WT) **(A)** and *Fam161a* knock-in (KI) **(B)** mouse retina with primary rabbit anti-Fam161a antibody (red), antiblue Opsin (green), and 4′,6-diamidino-2-phenylindole (DAPI) (blue). Immunohistochemistry (IHC) stain; original magnification, × 40. Scale bar = 20 μm. Green arrowheads point toward the cilia in blue cones. **C, D,** IHC stain; higher magnification, × 100 presented in **A** and **B**. Double staining of 1-month-old WT (**E**) and *Fam161a* KI (**F**) mouse retina with primary rabbit anti-Fam161a antibody (red), antired/green Opsin (green) and DAPI (blue). Green arrowheads point toward the cilia in red/green cones. **G, H,** IHC stain; higher magnification, × 100 presented in **E** and **F**. Cones and Fam161a expression in cone subtypes in KI mice at the age of 6 months. KI mouse retina stained with anti-Peanut Agglutinin (PNA) (**I**), anti blue Opsin (**J**) and anti red/green Opsin (**K**). Nuclei in all panels stained with DAPI (blue). IHC stain; original magnification, × 40. Scale bar = 20 μm. **L,** Average number of different cone types in the retina of 1-month-old and 6-month-old WT and KI mice. Cells were counted on each 1 of the single images, taken in a magnification of × 40, from different mice. Different colors represent different mice strains, and different tones of the color represent different ages. Error bars show standard error of the mean. Only significant results in *t* test are shown, and marked as *P* < 0.05 (∗), *P* < 0.01 (∗∗). IS = inner segments; INL = inner nuclear layer; ONL = outer nuclear layer; OPL = outer plexiform layer; OS = outer segments; RPE = retinal pigment epithelium.

Subsequently, double IHC labeling was performed to identify Fam161a expression in different types of cones. This revealed that Fam161a is expressed in the majority of both blue and red-green mouse cones in 1-month-old WT retina (Fig 6A, C, E, G). Both cone types were evident in 1-month-old KI retina; however, only a few cells showed faint expression of Fam161a (Fig 6B, D, F, H).

### Effect of p.Arg512∗ on Photoreceptor Survival

To better understand the pattern of photoreceptor degeneration, we examined the retinas of 1 and 6-months-old WT and KI mice using different antibodies for different cone types. The timepoint of 6 months was chosen because total retinal thickness is significantly reduced at this age, but the ONL is not yet totally degenerated. We observed that in KI retina, many cones are still evident at the age of 6 months as shown by anti-PNA staining which labels the cone OS (Fig 6I). Quantification of the total amount of PNA-positive cones at different ages in KI mice revealed some reduction along a period of 6 months, but this reduction was not statistically significant (Fig 6L). Specific staining of blue cones with anti blue opsin antibody (Fig 6J) as well as red/green cones with anti red/green opsin antibody (Fig 6K) revealed that both types of cones are still present at this age. Statistical analysis showed that both the reduction in red/green cones (*P* = 0.008) and blue cones (*P* = 0.04) was statistically significant in KI mice at 6 compared with 1 month of age. The only significant difference between WT and KI mice was observed in the number of red/green cones at the age of 6 months (*P* = 0.013). The remaining comparisons were not statistically significant (Fig 6L). The findings show that cone photoreceptor loss occurs rather late in this model.

## Discussion

In the current study, we characterized structural and functional progression of RD in a newly generated *Fam161a* KI mouse model, harboring the p.Arg523∗ founder nonsense pathogenic variant that is common among patients of Jewish origin with RP. This pathogenic variant results in an abnormal mRNA molecule that contains a PTC that can either produce a truncated protein or be degraded by the nonsense-mediated mRNA decay surveillance system. Using RT-PCR, we were able to show that the 2 major *Fam161a* transcripts (with or without the alternatively spliced exon #4) are present in KI mice retina and in WT retina, indicating that nonsense mediated decay does not degrade the abnormal mRNA molecules and therefore a truncated protein is likely to be produced.

This is supported by IHC studies showing positive anti-Fam161a staining in KI retinas, albeit with a reduced and abnormal pattern as compared to WT. The antibody we used is polyclonal and can potentially recognize both truncated and full-length proteins, and it should be noted that an extremely low level (0.01%–1%^19^^,^^35^) of full-length proteins (that include a missense variant) might be produced by spontaneous TR. Thus, it is possible that the relatively slow disease progression in this mouse model is because of residual function of the truncated protein.

In further detail, previous reports^8^^,^^9^ indicated that Fam161a is expressed in various retinal cells, including the OPL and photoreceptors (mainly in the cilia). Using IHC staining, we showed a similar pattern in the WT mouse retina. In the KI retina, however, faint and clumpy Fam161a expression is evident, indicating that some level of protein translation still occurs, but at much lower levels and with different distribution when compared with WT. The expression level of Fam161a in the OPL does not seem to differ between the KI and WT retina. The localization of Fam161a in the KI retina is not restricted to the cilia but can also be found in the IS of the photoreceptors, mainly in the photoreceptor region next to the ELM and at the base of the ISs. This distribution suggests that the abnormal protein is unable to reach the cilia and properly organize there, either because of misfolding of the mutant protein or because a protein localization sequence that might be located at the C-terminal region is missing (although such a sequence has not yet been identified). Interestingly, low levels of aggregated Fam161a protein were identified along the cilia of rods and both types of mouse cones. It is possible that some of the truncated protein might reach the cilia and its partial function might play a role in slowing down disease progression, as was previously described in a rat model of RD.^36^

An interesting insight might be obtained from comparison of the onset and severity of the retinal phenotype of the KI mouse model we describe here to the 2 previously reported *Fam161a* mouse models: *Fam161a*^GT/GT^ and the KO *Fam161a*^*tm1b/tm1b*^ model that we recently reported.^12^^,^^13^ Elaborate characterization of retinal structure and function in the KI model between 1 and 21 months of age revealed a relatively late and slow RD. OCT and histologic analyses of the KI retina at the age of 3 months showed mild loss, whereas in the 2 other models of *Fam161a*, RD was already evident by OCT at the age of 1 month. In addition, retinal function analysis using optomotor tracking (measuring VA) and ERG also showed later disease onset in the KI model. Moreover, the rate of functional loss is much slower in the KI model than that in the 2 other *Fam161a* models. The structural and functional parameters measured were well correlated and all showed very slow disease progression until the age of 12 months, with more rapid degeneration evident between 15 and 21 months, at which time no photoreceptor activity could be measured in the KI retina and only a few photoreceptor nuclei were seen in histology. In comparison, the other 2 published models manifested severe degeneration by the age of 6 to 8 months (Fig S4, available at https://www.ophthalmologyscience.org).^12^^,^^13^ This is emphasized by retinal morphometry showing that between 1 and 18 months (Fig 4A–C, Table 1, and Fig S4, available at https://www.ophthalmologyscience.org), ONL thickness was reduced by ∼ 90% and total retinal thickness by ∼ 50% in KI mice, whereas the reported *Fam161a* models reached this stage at the age of 6 to 8 months.^12^^,^^13^ Similarly, while VA in the KI model gradually decreased from 1 to 21 months of age with 50% reduction at 15 months, KO mice had already reached this VA level at the age of 6 months.

Although they differ in age of onset and in rate of progression, in all *Fam161a* models the degenerative process initiates and mainly involves the photoreceptor layer, ^12^^,^^13^ with a faster decrease in scotopic versus photopic ERG responses. This indicates a rod>cone pattern of degeneration, thus recapitulating disease course in biallelic *FAM161A* human patients.^13^

We previously conducted a clinical analysis of a large set of biallelic *FAM161A* patients harboring 1 of 3 possible genotypes: homozygous for a frameshift, homozygous for the nonsense pathogenic variant (p.Arg523∗), or compound heterozygotes.^14^ We reported that on average, homozygotes for p.Arg523∗ show a statistically significant earlier loss of best-corrected VA as compared with the other 2 genotypes. A similar pattern, although it did not reach a statistically significant value, was also detected in the analysis of cone ERG amplitudes. Interestingly, the opposite trend is seen in mice, in which more severe retinal disease is evident in the KO mouse model,^13^ which is equivalent to the homozygous frameshift group.

The 2 founder pathogenic variants identified in Jewish patients are located within the same exon, exon #3, and are 212bp apart: frameshift (p.Thr452Serfs∗3) and nonsense (p.Arg523∗). Assuming no effect of the nonsense mediated decay system on these pathogenic variants (as shown in human fibroblasts^24^ and the mouse retina), both are expected to result in truncated proteins that will not include the highly conserved sequence encoded by the alternatively-spliced exon, as well as the conserved C-terminal protein domain (UPF0564). These truncated proteins are therefore likely to be nonfunctional; however, one cannot exclude the possibility of partial function or even potential toxicity.

The comparison of the retinal phenotype of *FAM161A* human patients and *Fam161a* mouse models revealed some interesting similarities that attest to the degenerative process. OCT scans indicate retinal thickness in vivo and enable serial follow-up. In *FAM161A-*RP patients, retinal thickness was reduced with age with the main reduction found in the ONL.^14^ These results are in line with those obtained in the *Fam161a* mouse models where progressive thinning of the total retina, mainly the ONL, were observed. Fundus autofluorescence images documented a hyper-autofluorescent ring around the fovea in the vast majority of patients, including at young ages (second decade of life), along with hypo-autofluorescent spots in the mid periphery. In both mouse models, hypo-autofluorescent and hyper-autofluorescent spots also appear throughout the fundus over time. These findings indicate that the retina undergoes a degenerative process and might indicate the recruitment of microglial cells to eliminate degenerated or damaged photoreceptors.^37^^,^^38^

To conclude, in this study, we developed and characterized the course of retinal disease in KI mice with a homozygous nonsense pathogenic variant similar to a common human one. Affected mice show low levels and abnormal distribution of Fam161a expression. The pattern of photoreceptor degeneration is similar to that seen in human *FAM161A*-RP patients with rod involvement followed by cone degeneration, as evident from structural and functional examinations of the retina. Furthermore, this model is significantly slower than other commonly used mouse models of RD, such as rd1 and rd10 and is also slower than the 2 other previously described *Fam161a* mouse models. This slow degeneration, which in many ways recapitulates the human disease, allows a wider window for testing novel therapeutic interventions, including gene augmentation therapy, RNA editing therapies, or treatment with TRIDs, which can suppress the nonsense pathogenic variant resulting in production of a full-length protein. Our aim is to use this model to examine safety and possible efficacy of such potential treatments, which can serve as a first step toward future application in patients manifesting *FAM161A*-associated RD.
