# Supplementary material for: Retinal Structure and Function in a Knock-in Mouse Model for the FAM161A-p.Arg523∗ Human Nonsense Pathogenic Variant
Source: Ophthalmol Sci. 2022 Oct 3;3(1):100229. doi: 10.1016/j.xops.2022.100229 (PMC9676433; doi:10.1016/j.xops.2022.100229)
Supplement: Supplementary Figure S1 [file mmc1.pdf]

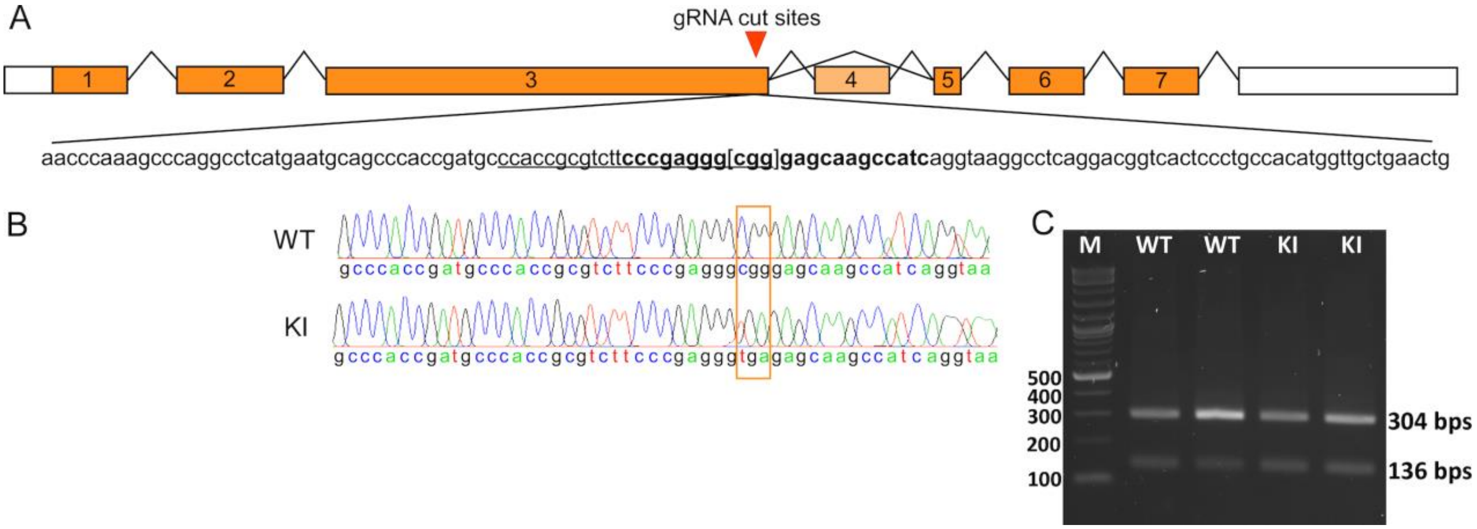

Supplementary Figure S1: Gene structure and location of the KI mutation. A. Schematic representation of mouse *Fam161a*. Orange blocks represents the exons of the gene, with the exon number. Light orange represents alternative exon #4. The targeted sequence is detailed below, forward guide RNA is underlined, and reverse guide RNA is shown by bold letters. The targeted sequence in square brackets. **B.** Chromatogram of WT mouse and *Fam161a* KI mouse depicting the targeted area. The changed codon is marked with an orange rectangle. **C.** RT-PCR analysis on *Fam161a* KI and WT mouse retina using primers located in exons 3 and 5. The lower band (136 bps) does not contain exon 4 while the upper band (304 bps) contains this alternatively spliced exon.
