# Supplementary material for: Retinal Structure and Function in a Knock-in Mouse Model for the FAM161A-p.Arg523∗ Human Nonsense Pathogenic Variant
Source: Ophthalmol Sci. 2022 Oct 3;3(1):100229. doi: 10.1016/j.xops.2022.100229 (PMC9676433; doi:10.1016/j.xops.2022.100229)
Supplement: Supplementary Figure S2 [file mmc2.pdf]

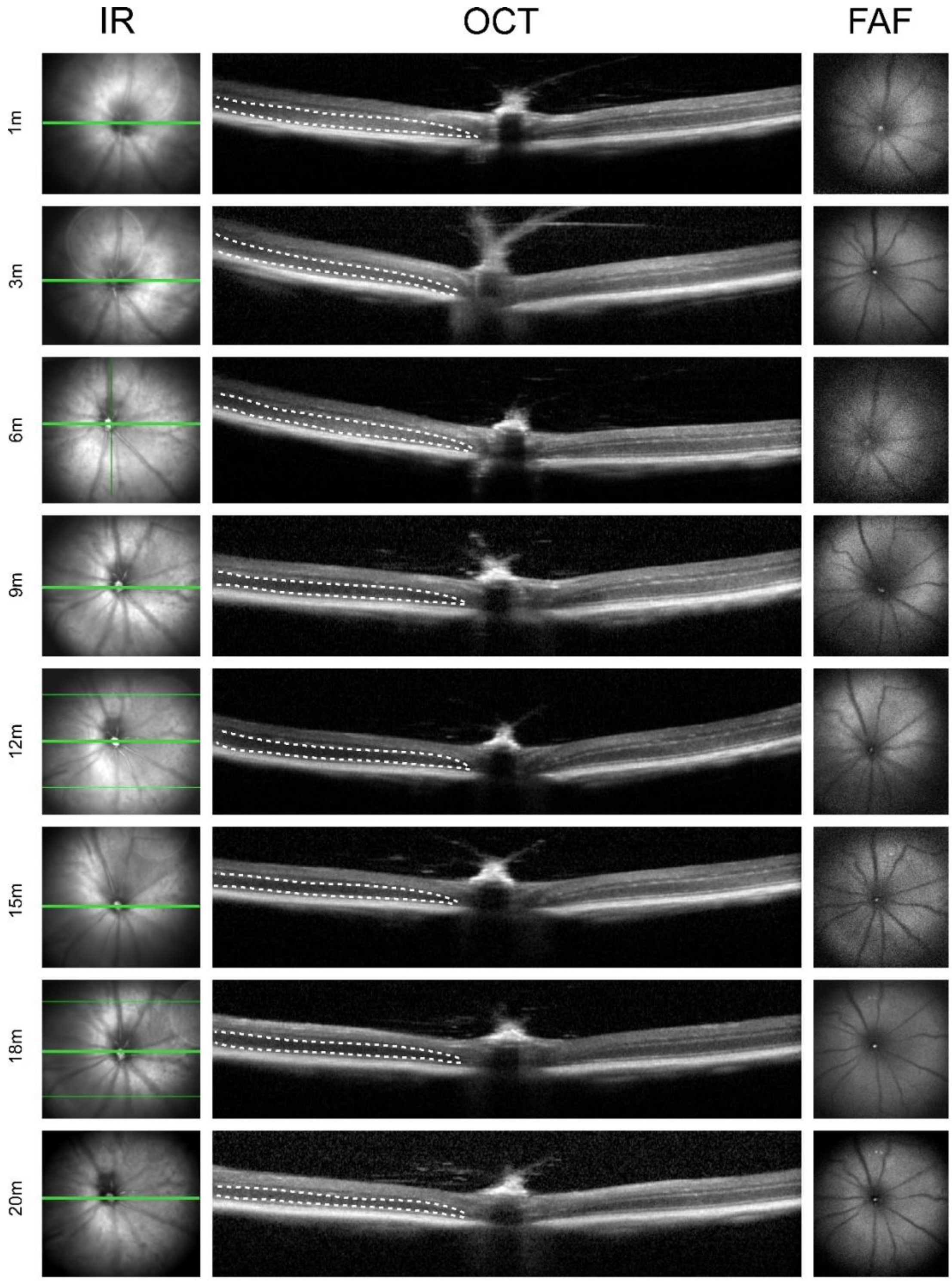

Supplementary Figure S2: IR, OCT and FAF imaging of control WT mice at different ages that correspond to the imaging of *Fam161a* KI mice presented in Figure 3 of the main manuscript. WT mouse retinas at 1, 3, 6, 9, 12, 18 and 20 month/s of age is presented. The outer nuclear layer which represents the photoreceptors is marked by dotted lines.
