# Supplementary material for: Retinal Structure and Function in a Knock-in Mouse Model for the FAM161A-p.Arg523∗ Human Nonsense Pathogenic Variant
Source: Ophthalmol Sci. 2022 Oct 3;3(1):100229. doi: 10.1016/j.xops.2022.100229 (PMC9676433; doi:10.1016/j.xops.2022.100229)
Supplement: Supplementary Figure S3 [file mmc3.pdf]

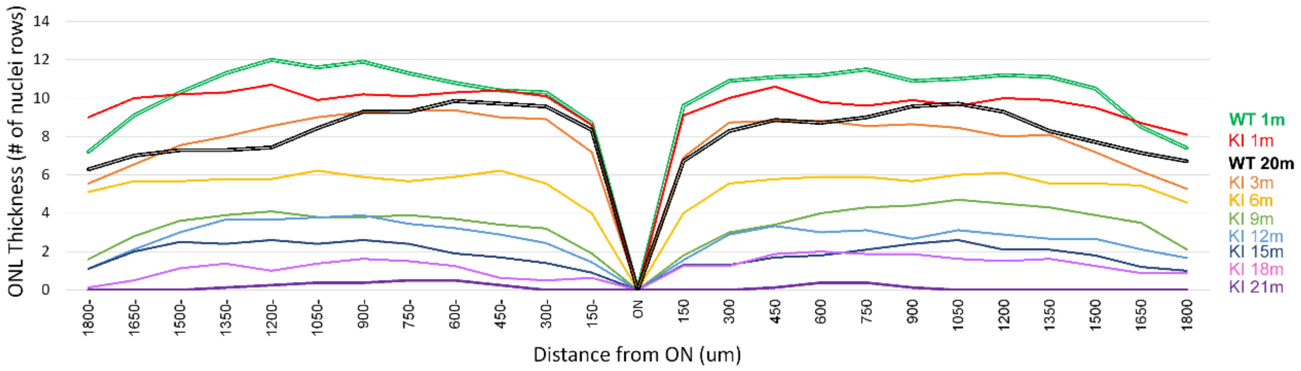

Supplementary Figure S3: ONL thickness in *Fam161a* KI and WT mice.

To quantify ONL structure and disease progression, number of rows of nuclei in the ONL was counted in150µmsteps from the optic nerve towards the nasal and temporal periphery. WT retinas at the ages of 1 and 20 months are compared to *Fam161a* KI between 1-21 months of age. Image-J software was used.
