# Supplementary material for: Retinal Structure and Function in a Knock-in Mouse Model for the FAM161A-p.Arg523∗ Human Nonsense Pathogenic Variant
Source: Ophthalmol Sci. 2022 Oct 3;3(1):100229. doi: 10.1016/j.xops.2022.100229 (PMC9676433; doi:10.1016/j.xops.2022.100229)
Supplement: Supplementary Figure S4 [file mmc4.pdf]

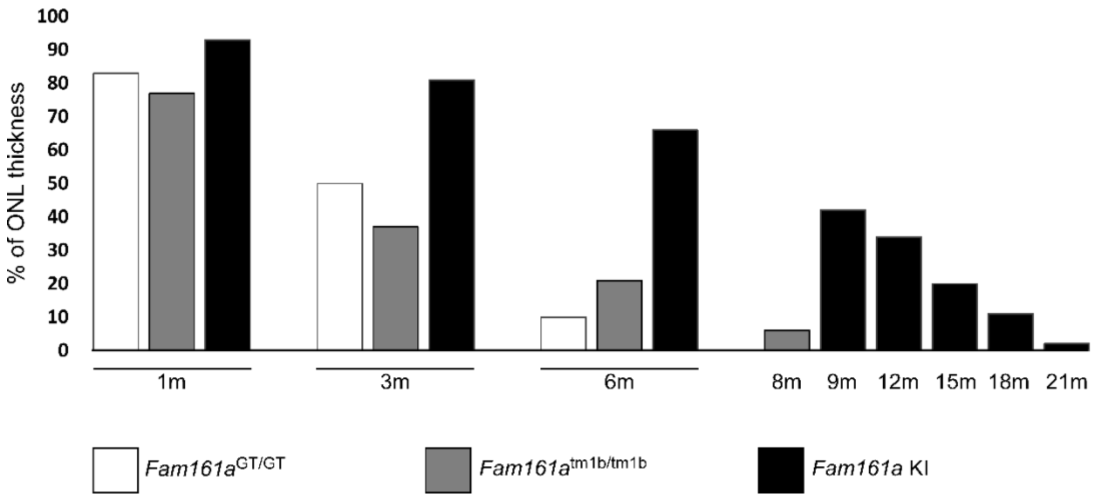

Supplementary Figure S4: Comparative morphometric illustration of outer nuclear layer (ONL) thickness loss over time in three *Fam161a* mouse models; *Fam161a*$^{GT/GT}$ (white) [Karlstetter *et al*., 2014], *Fam161a*$^{tm1b/tm1b}$ (grey) [Beryozkin *et al*., 2021] and the current study model *Fam161a* KI (black). Comparison of average ONL thickness between the different mouse models for each timepoint is presented as percentage compare to 1 month WT retina as a control. ONL thickness deteriorates over time in all models, with ONL thickness loss being much slower in the *Fam161a* KI model. ONL thickness was quantified in H&E stained histological retinal sections passing through the optic nerve.
