# Supplementary material for: Retinal Structure and Function in a Knock-in Mouse Model for the FAM161A-p.Arg523∗ Human Nonsense Pathogenic Variant
Source: Ophthalmol Sci. 2022 Oct 3;3(1):100229. doi: 10.1016/j.xops.2022.100229 (PMC9676433; doi:10.1016/j.xops.2022.100229)
Supplement: Supplementary Table S1 [file mmc5.pdf]

Supplementary Table S1: Primer sequences used in this study

**Reaction name**		**Forward primer**	**Revers primer**	Length (bp)
Mutations specific for 6N and 6J strains	CRB1 ex9	gcacaatagagattggaggc	tgtctacatccacctcacag	386
	RPE65 ex13	catatgctagtcaagtaaagcag	cccaacccctttcctaatagag	457
	GNAT2 ex5	accgatgccaccttcttttt	tgctgtgagacctgagatgc	265
	PDE6B ex13	ggctctgatatggtgctgtg	aaagactcaccctaaggacgc	453
	PDE6B ex16	gacagagtaggccctgagagtc	cctgggattagggaggtctg	655
	PDE6B insertion	tgacaattactccttttccctcagtctg	gtaaacagcaagaggctttattgggaac<br>tacccacccttcctaatttttctcacgc	398<br>587
Genotyping of the strain	WT and KI	ttgcagacgtttcagggacccc	gacttaacagggcataattcaaaggc	659
RT-PCR Exon 3-5	Identification of the expressed isoform	gtctcctaggcgcaagtcac	tctcatcctggccttttcacctc	304<br>136
